# Supplementary material for: Enhancing the land use efficiency of low-land rice (Oryza sativa L.)—Grass pea (Lathyrus sativus L.) additive series relay intercropping in North-Western Ethiopia: A farmer’s indigenous knowledge
Source: PLoS One. 2023 Jul 6;18(7):e0281410. doi: 10.1371/journal.pone.0281410 (PMC10325089; doi:10.1371/journal.pone.0281410)
Supplement: S1 File — (DOCX) [file pone.0281410.s001.docx]

SI 1: Data set up for normal statistical analysis (grass pea)

| SPGP | PM | Rep | PL | PH | NSPP | ET | NET | FG | UG | NSEP | TGW | DBM | GY | HI |
| --- | --- | --- | --- | --- | --- | --- | --- | --- | --- | --- | --- | --- | --- | --- |
| 1 | 1 | 1 | 13.9 | 104.4 | 10.2 | 8.9 | 2 | 120 | 9.8 | 144 | 24.8 | 129.6 | 50 | 38.5 |
| 1 | 1 | 2 | 13.2 | 108.8 | 10.2 | 8.3 | 2 | 123.8 | 9.2 | 144 | 23.5 | 140.7 | 49.62 | 35.3 |
| 1 | 1 | 3 | 13.6 | 105.6 | 10.8 | 8.4 | 2 | 121.8 | 11 | 145.9 | 23.4 | 129.6 | 48.88 | 37.7 |
| 1 | 2 | 1 | 12.8 | 107.6 | 9.6 | 7 | 2.9 | 123.5 | 12.4 | 147.4 | 23.3 | 144.4 | 49.25 | 34.1 |
| 1 | 2 | 2 | 13.4 | 106.2 | 10.4 | 7.5 | 2.2 | 124.9 | 11 | 150.1 | 23.6 | 133.3 | 48.14 | 36.1 |
| 1 | 2 | 3 | 14.8 | 104.8 | 10.6 | 8.8 | 2.4 | 122 | 12.2 | 150.5 | 23.2 | 141.6 | 48.14 | 33.9 |
| 1 | 3 | 1 | 13 | 103.6 | 9.8 | 7.8 | 2.5 | 122 | 11.2 | 148 | 23.6 | 103.7 | 40 | 38.5 |
| 1 | 3 | 2 | 13.4 | 105.8 | 10.8 | 8.6 | 2 | 120 | 12.8 | 146 | 23.2 | 144.4 | 49.62 | 34.3 |
| 1 | 3 | 3 | 14.8 | 104.2 | 10.4 | 8 | 2.5 | 124.7 | 9.2 | 143 | 23.3 | 125.9 | 48.51 | 38.5 |
| 1 | 4 | 1 | 13 | 105 | 9 | 8 | 2.4 | 121 | 12 | 148 | 24 | 111.1 | 43.33 | 39 |
| 1 | 4 | 2 | 13 | 104.5 | 11 | 7 | 2 | 121.3 | 9.2 | 148 | 24.7 | 133.3 | 51.11 | 38.3 |
| 1 | 4 | 3 | 14 | 108 | 9.6 | 8.1 | 1.9 | 120 | 9 | 146 | 24.8 | 118.5 | 46.29 | 39 |
| 2 | 1 | 1 | 12 | 106.4 | 10 | 8 | 2.5 | 123.4 | 11.7 | 143 | 24.5 | 125.9 | 47.77 | 37.9 |
| 2 | 1 | 2 | 13.8 | 104.6 | 9.8 | 6.8 | 2 | 122 | 9.2 | 150 | 23.8 | 125.9 | 50 | 39.7 |
| 2 | 1 | 3 | 13.4 | 107.2 | 10.6 | 7.5 | 2 | 123 | 11.4 | 149.4 | 23.4 | 144.4 | 57.77 | 40 |
| 2 | 2 | 1 | 12.2 | 103.4 | 8.4 | 7.2 | 2.3 | 124 | 9 | 144.9 | 23.6 | 144.4 | 56.66 | 39.2 |
| 2 | 2 | 2 | 13 | 106.4 | 9.6 | 7 | 2 | 124 | 9.6 | 147 | 24.1 | 133.3 | 50.74 | 38 |
| 2 | 2 | 3 | 13.2 | 108.6 | 10.2 | 7.5 | 1.9 | 122.9 | 9.8 | 148.1 | 23.8 | 137 | 52.22 | 38.1 |
| 2 | 3 | 1 | 12 | 105.5 | 9 | 7.9 | 3 | 122.8 | 9.2 | 150 | 23.4 | 125.9 | 48.51 | 38.5 |
| 2 | 3 | 2 | 13 | 104.2 | 10.4 | 8 | 2.4 | 124.7 | 12 | 149.8 | 23 | 122.2 | 47.03 | 38.4 |
| 2 | 3 | 3 | 13.8 | 106.4 | 9.6 | 7.9 | 2.5 | 124.6 | 9 | 151.2 | 23.9 | 140.7 | 54.07 | 38.4 |
| 2 | 4 | 1 | 12.6 | 104 | 8.2 | 7.4 | 1.9 | 123 | 11.7 | 147.4 | 24.2 | 125.9 | 49.25 | 39.1 |
| 2 | 4 | 2 | 13 | 104.8 | 9.8 | 7.6 | 2 | 122 | 13.2 | 145.9 | 23.6 | 122.2 | 48.14 | 39.3 |
| 2 | 4 | 3 | 13.5 | 108.2 | 9.8 | 8.5 | 2.2 | 120 | 12.8 | 144.9 | 24.3 | 129.6 | 50.37 | 38.8 |
| 3 | 1 | 1 | 12.6 | 106.7 | 10 | 7.2 | 3 | 123 | 12 | 144.9 | 23.1 | 118.5 | 45.55 | 38.4 |
| 3 | 1 | 2 | 13.5 | 103.8 | 10 | 7.4 | 2 | 123 | 9.2 | 149.9 | 24.5 | 122.2 | 48.51 | 39.6 |
| 3 | 1 | 3 | 13.3 | 104.4 | 8.8 | 7.5 | 2.4 | 125 | 9.4 | 149.4 | 24.5 | 118.5 | 47.03 | 39.6 |
| 3 | 2 | 1 | 13.2 | 106.8 | 10 | 8 | 1.9 | 121.9 | 12.2 | 149.9 | 24.3 | 122.2 | 47.4 | 38.7 |
| 3 | 2 | 2 | 13.5 | 106.7 | 9.6 | 7.4 | 1.8 | 125 | 12.9 | 147 | 23.3 | 114.8 | 44.81 | 39 |
| 3 | 2 | 3 | 13.2 | 105.6 | 10.2 | 8.6 | 2 | 123.5 | 10.4 | 151 | 24.7 | 144.4 | 56.29 | 38.9 |
| 3 | 3 | 1 | 13.6 | 107.4 | 10.6 | 7.8 | 2.1 | 123.7 | 9.9 | 150.4 | 24.5 | 133.3 | 51.85 | 38.8 |
| 3 | 3 | 2 | 13.2 | 106.6 | 10.8 | 7.9 | 2.8 | 122 | 12.4 | 144.2 | 23.6 | 114.8 | 44.07 | 38.3 |
| 3 | 3 | 3 | 12.5 | 108.4 | 8.8 | 7.6 | 2.9 | 125.8 | 12 | 150 | 23.4 | 137 | 53.33 | 38.9 |
| 3 | 4 | 1 | 12.7 | 106.5 | 10 | 7.6 | 2 | 124 | 11.6 | 146 | 24.2 | 122.2 | 48.51 | 39.6 |
| 3 | 4 | 2 | 12.5 | 107.2 | 9.4 | 7.8 | 2.9 | 123 | 9.4 | 145 | 24.1 | 114.8 | 44.07 | 38.3 |
| 3 | 4 | 3 | 12.8 | 107.4 | 10.4 | 8.3 | 2.6 | 122 | 12.7 | 144.9 | 24.1 | 118.5 | 45.18 | 38.1 |
| 4 | 1 | 1 | 12.2 | 106.2 | 10 | 7.2 | 1.9 | 122.4 | 10.6 | 142.4 | 24 | 125.9 | 50.37 | 40 |
| 4 | 1 | 2 | 13.2 | 105.9 | 9 | 8.9 | 2 | 120.9 | 13 | 148.8 | 23 | 144.4 | 56.66 | 39.2 |
| 4 | 1 | 3 | 8 | 108.8 | 10 | 7 | 2.6 | 124 | 11.8 | 144.6 | 24.7 | 114.8 | 45.92 | 40 |
| 4 | 2 | 1 | 12.6 | 107.2 | 9.8 | 7.9 | 2.5 | 122.7 | 11 | 148 | 23.5 | 122.2 | 47.77 | 39 |
| 4 | 2 | 2 | 13.6 | 104.7 | 8.8 | 7.9 | 2 | 120.2 | 9 | 146.9 | 23.4 | 140.7 | 54.44 | 38.6 |
| 4 | 2 | 3 | 13.7 | 106 | 10.6 | 7.8 | 2.6 | 120.8 | 14 | 144 | 23.7 | 111.1 | 43.7 | 39.3 |
| 4 | 3 | 1 | 13.4 | 107 | 9.4 | 7.5 | 2.6 | 121 | 12.6 | 143.8 | 23.7 | 118.5 | 41.85 | 35.3 |
| 4 | 3 | 2 | 13.2 | 105.6 | 9 | 7.5 | 1.6 | 121.7 | 13.4 | 144 | 24.2 | 129.6 | 43.7 | 39.9 |
| 4 | 3 | 3 | 13 | 104.5 | 9.2 | 9 | 2.6 | 125.7 | 14 | 145 | 23.4 | 144.4 | 41.85 | 33.7 |
| 4 | 4 | 1 | 12.8 | 105.6 | 9.2 | 8 | 1.8 | 124.8 | 9.8 | 145.5 | 23.2 | 118.5 | 45.18 | 38.1 |
| 4 | 4 | 2 | 13.8 | 108.3 | 10.8 | 8.5 | 2 | 123.2 | 13 | 148.5 | 23.5 | 125.9 | 48.51 | 38.5 |
| 4 | 4 | 3 | 13.2 | 105.6 | 10.2 | 9 | 1.8 | 123 | 13.8 | 145.9 | 23.6 | 129.6 | 49.62 | 38.2 |
