# Supplementary material for: Enhancing the land use efficiency of low-land rice (Oryza sativa L.)—Grass pea (Lathyrus sativus L.) additive series relay intercropping in North-Western Ethiopia: A farmer’s indigenous knowledge
Source: PLoS One. 2023 Jul 6;18(7):e0281410. doi: 10.1371/journal.pone.0281410 (PMC10325089; doi:10.1371/journal.pone.0281410)
Supplement: Attachment — Submitted filename: Reviewer Commen-WPS Office.docx [file pone.0281410.s003.docx]

**Reviewer Comments**

1) Grammetical errors need to be corrected.

2)Introduction part needs to be improvised accrording to the title of the research paper.

3)The different planting methods and patterns can be displayed in a table form in the materials and methods.

4) Results are highlighted only for some parameters. May explained in detail.

5)Discussion part need to be modified more prescicely.

6)Conclusion doesn't highlight the whole research. It need to be revised.
