# Supplementary material for: Enhancing the land use efficiency of low-land rice (Oryza sativa L.)—Grass pea (Lathyrus sativus L.) additive series relay intercropping in North-Western Ethiopia: A farmer’s indigenous knowledge
Source: PLoS One. 2023 Jul 6;18(7):e0281410. doi: 10.1371/journal.pone.0281410 (PMC10325089; doi:10.1371/journal.pone.0281410)
Supplement: Attachment — Submitted filename: Response to Reviewers.docx [file pone.0281410.s004.docx]

1) Grammetical errors need to be corrected.

Response: fully checked ad corrected y grammar checker

2) Introduction part needs to be improvised accrording to the title of the research paper.

Response: Updated Based on the comment

3) The different planting methods and patterns can be displayed in a table form in the materials and methods.

Response: Clearly descried IN the document

Table 2. Treatment combination used in additive series relay intercropping of grass pea with rice

| **Trt No.** | **SPGP (%)** | **SA (R: GP)** | **TCS** |
| --- | --- | --- | --- |
| **1** | 25 | 1:1 | Row intercropping |
| **2** | 25 | 2:1 | Row intercropping |
| **3** | 25 | 3:1 | Row intercropping |
| **4** | 25 | MRI | Mixed intercropping |
| **5** | 50 | 1:1 | Row intercropping |
| **6** | 50 | 2:1 | Row intercropping |
| **7** | 50 | 3:1 | Row intercropping |
| **8** | 50 | MRI | Mixed intercropping |
| **9** | 75 | 1:1 | Row intercropping |
| **10** | 75 | 2:1 | Row intercropping |
| **11** | 75 | 3:1 | Row intercropping |
| **12** | 75 | MRI | Mixed intercropping |
| **13** | 100 | 1:1 | Row intercropping |
| **14** | 100 | 2:1 | Row intercropping |
| **15** | 100 | 3:1 | Row intercropping |
| **16** | 100 | MRI | Mixed intercropping |
| **17** | Sole rice | - | Row planting |
| **18** | Sole grass pea | - | Broadcasting |

Trt No., treatment number; SPGP (%), seed proportion of grass pea; SA, spatial arrangement; TCS, type of cropping system; GP, Grass pea; R, rice; **R: GP, rice to grass pea seed proportions;** MRI, mixed relay intercropping.

4) Results are highlighted only for some parameters. May explained in detail.

Response: Yes, because the main objectives of this work is to evaluate the lad use efficiency usig different competition indices

5) Discussion part need to be modified more precisely.

6) Conclusion doesn't highlight the whole research. It need to be revised.

Response: The study confirmed that seed proportion of grass pea and spatial arrangement in additive series relay intercropping of grass pea with low-land rice had no significant effect on rice. The highest grain yield of grass pea was obtained when 25% SPGP was relay intercropped with rice in 1:3 SA. The LER and ATER values of all intercropping systems were sufficiently greater than one, indicating that growing the component crops in an intercropping was advantageous than growing the component crops individually. The rice equivalent yield for different rice-grass pea relay intercropping systems was higher when 25% of SPGP was relay intercropped with rice in 1:3. Although, the monetary advantage index was higher in rice-grass pea relay intercropping when 75% of SPGP was relay intercropped with 1:3 SA, the monetary advantage index (MAI) values were positive in all intercropping systems. The highest net benefit was recorded when 50% SPGP was relay intercropped with rice in 1:3 SA. In conclusion, maximum production efficiency in terms of TLOY and land use efficiency, NB, MRR, and positive MAI with lower CR was obtained when 50% SPGP was intercropped with the full seed rate of rice in 1:3 SA, indicating that this cropping system is a far better production system as compared to what farmers currently use (mixed intercropping system). Thus, this mixture seems to contribute to the development of sustainable crop production systems with limited external inputs. The following research gaps were suggested for further research: (i) the experiment needs to be repeated across locations and years as the experiment was conducted in a specific area and in one growing season; (ii) the effect of grass pea on soil fertility needs to be investigated as rice (the main crop) and grass pea (a supplementary crop) relay intercropping is the dominant cropping system in the study area; (iii) rice intercropping with other staple legume crops needs to be tested to intensify the production efficiency and profitability of the cropping system.

**Comments to the Author**

1. Is the manuscript technically sound, and do the data support the conclusions?

The manuscript must describe a technically sound piece of scientific research with data that supports the conclusions. Experiments must have been conducted rigorously, with appropriate controls, replication, and sample sizes. The conclusions must be drawn appropriately based on the data presented.

Reviewer #1: Partly

Reviewer #2: Yes

Response:

2. Has the statistical analysis been performed appropriately and rigorously?

Reviewer #1: Yes

Reviewer #2: Yes

3. Have the authors made all data underlying the findings in their manuscript fully available?

The [PLOS Data policy](http://www.plosone.org/static/policies.action#sharing) requires authors to make all data underlying the findings described in their manuscript fully available without restriction, with rare exception (please refer to the Data Availability Statement in the manuscript PDF file). The data should be provided as part of the manuscript or its supporting information, or deposited to a public repository. For example, in addition to summary statistics, the data points behind means, medians and variance measures should be available. If there are restrictions on publicly sharing data—e.g. participant privacy or use of data from a third party—those must be specified.

Reviewer #1: Yes

Reviewer #2: Yes

4. Is the manuscript presented in an intelligible fashion and written in standard English?

PLOS ONE does not copyedit accepted manuscripts, so the language in submitted articles must be clear, correct, and unambiguous. Any typographical or grammatical errors should be corrected at revision, so please note any specific errors here.

Reviewer #1: No

Reviewer #2: No

Response: updated

5. Review Comments to the Author

Please use the space provided to explain your answers to the questions above. You may also include additional comments for the author, including concerns about dual publication, research ethics, or publication ethics. (Please upload your review as an attachment if it exceeds 20,000 characters)

Reviewer #1: he entitled manuscript “Enhancing additive series relay intercropping of grass pea (Lathyrus sativus L.) with low land rice (Oryza sativa L.) in North-Western Ethiopia: A farmer’s indigenous knowledge”.
The submitted manuscript emphasised that seed proportion of grass pea and spatial arrangement had no significant effect on rice crop in additive series relay intercropping of grass pea with low land rice.
However, the present study trial has also been conducted in one season in single location in Ethiopia, Therefore, the reliability of the results is doubtful and cannot make conclusion on the basis of it. Therefore, it is suggested that at least Two year and multi-location data should be included in the present submitted manuscript. Apart from this, the present manuscript need to rectify the grammatical as well as typological mistakes.

**Response: further research is suggested ad the manuscript was updated grammatical**

Observations:

1. Please check the sentence “Banik et al. [53], who observed that the chick pea yield in wheat-check pea mixture significantly lower than sole cropped check pea.”

Response: This result contradicted the results of Banik et al. [53], who observed that the chickpea yield in a wheat-chickpea mixture was significantly lower than that of a solely cropped chickpea.
2. Please correct the words Results “Results revealed that the CR of rice was greater”
3. Too lengthy sentences and not clear “Results revealed that partial land equivalent ratio (PLER) of rice and grass pea in all cropping systems were greater than 0.5 (Figure 4 ) indicating that there was null or very low growth resource competition between the component crops during their co-growing period which supports the CR values.

Response: corrected based on the comment

Reviewer #2: The article needs to be modified according to the comments ( Please find the attachment )
The introduction and discussion part need to be improvised with the adsition of related articles and findings.

Response: corrected based on the comment
